# Familial Mediterranean Gene (MEFV) Mutation in Parents of Children with Familial Mediterranean Fever: What Are the Exceptions?

**DOI:** 10.1155/2018/1902791

**Published:** 2018-10-01

**Authors:** Leila Shahbaznejad, Sayed-Reza Raeeskarami, Raheleh Assari, Abbas Shakoori, Hamidreza Azhideh, Yahya Aghighi, Fatemeh Tahghighi, Vahid Ziaee

**Affiliations:** ^1^Children's Medical Center, Pediatrics Center of Excellence, Tehran, Iran; ^2^Department of Pediatrics, Mazandaran University of Medical Sciences, Sari, Iran; ^3^Pediatric Rheumatology Research Group, Rheumatology Research Center, Tehran University of Medical Sciences, Tehran, Iran; ^4^Department of Pediatrics, Tehran University of Medical Sciences, Tehran, Iran; ^5^Department of Medical Genetics, Tehran University of Medical Sciences, Tehran, Iran

## Abstract

**Objectives:**

Familial Mediterranean Fever (FMF) is one of the most prevalent periodic fever syndromes; MEFV, the responsible gene for the disease, is in the short arm of chromosome16. In the considerable count of the FMF patients, only one mutation is found in the MEFV and parents, who were the obligatory carriers for that mutation, were asymptomatic. The aim of this study was to evaluate these asymptomatic parents in regard to mutation in MEFV gene and similarity between parents and offspring patients.

**Methods:**

In this cross-sectional study, asymptomatic parents of FMF patients enrolled the study were referred to periodic fever clinic or pediatric rheumatology clinic of Tehran University of Medical Sciences. The patients should have at least one mutation in MEFV gene and none of them had any family history of autoinflammatory disease. Twelve mutations in MEFV gene were assessed in the parents by Vienna Lab FMF Strip Assay kit by MAS PCR/Reverse hybridization.

**Results:**

Forty-three patients and their parents participated in the study. Sixty-three percent (27) of patients were male. Onset of disease symptoms in 31 patients (72%) was before 4 years of old. Nine (21%) of the patients had homozygote, 16 (37%) compound heterozygote, and 17(40%) heterozygote for MEFV mutation; there was a case of complex alleles mutations (2%). M694V/M694V in 4 patients (9%) was the most homozygote genotype, and M694V/R761H in 4 (9%) and E148Q in 7 (16%) were the most compound heterozygote and heterozygote genotype, respectively. M694V, M680I, and E148Q were the most mutation in the parents. Overall, 41 patients had mutations similar to their parents' mutation, except 2 whose parents had no mutation, but a patient did.

**Conclusion:**

It seems that occurrence of new mutations in offspring is not prevalent among FMF patients and there are other reasons for different clinical presentation in similar mutation carriers. On the other hand, in ethnicities with high prevalence of FMF, new mutation in descendant may occur, infrequently.

## 1. Introduction

Familial Mediterranean fever (FMF) is one of the most frequent periodic febrile syndromes [1, 2]. Gene MEFV which was responsible for the disease was discovered in 1997, at the short arm of chromosome 16 and encode synthesis of pyrin/meranostrin [1, 2]. Today there are more than 300 variances in MEFV gene [2]. FMF is known as an autosomal recessive hereditary disease with different clinical presentation due to type of mutation, i.e., homozygote or heterozygote pattern. The other patients have not any known mutation or have only one mutation (heterozygote) [1, 2]. The patients are symptom-free between attacks, but it is notable that inflammatory reaction not only was active in each attack but also was between episodes in FMF patients. Also, in asymptomatic mutation carriers the inflammatory phenomenon has been demonstrated [3]. The most important complication of FMF is amyloidosis; when involving kidneys and other organs such as scrotum, an unfavorable prognosis is inevitable [1, 4]. The diagnosis of FMF is based on characteristic periodic complaints with fever, serositis, and a good clinical response to colchicine [1, 2].

To the best of our knowledge, there is no published study indicating mutations in the MEFV gene in asymptomatic parents of FMF patients, so we discovered MEFV gene of healthy and asymptomatic parents, who had an offspring suffering from FMF attacks and had at least one known mutation. We would like to know about mutation transmission in FMF patient population.

## 2. Methods and Materials

In this cross-sectional descriptive study, healthy parents of FMF patients had been evaluated about MEFV gene mutation. Each patient with two or more criteria of Yalcinkaya criteria [5] (fever, abdominal pain, chest pain, and arthritis) with exception of positive familial history of FMF in parents was included in our research. The exclusion criteria in each attack consisted of periodic oral and stomatitis aphthous lesion, periodic pharyngitis, periodic lymphadenitis (Marshal Syndrome), periodic noncharacteristic skin rashes, and CNS involvements. The patients were under observation at periodic fever clinic, children medical center (CMC), and Valiasr pediatric rheumatology clinic, Tehran University of Medical Sciences (TUMS). All of the involved patients should have at least one known mutation, and parents should not have any history for autoinflammatory disease.

At first, mutation of the present parent was evaluated; if it was negative or it was incompatible with the offspring mutation or the patient had more than one mutation, the other parent was also assessed. One milliliter of venous blood sample was needed to evaluate MEFV gene mutation with Vienna Lab FMF Strip Assay kit. The kit was equipped to assess 12 mutations in exons 2, 5, and 10 in MEFV gene, including mutations: E148Q, P369S, F479L, M680I(G/C), M680I(G/A), 1692del, M694V, M694I, K695R, V726A, A744S, and R761H. According to manufacturer's instruction, Multiplex PCR Amplification, Simultaneous Biotin Labeling, and reverse hybridization were performed, respectively. The MEFV gene assessment was done at genetic lab of cancer institute (TUMS).

The study was approved by TUMS ethics in research committee by number IR.TUMS.REC.1394.1855. All of the participants were aware of the process and free to engage the study and they should not pay any fee to the laboratory. All of them signed informed consent.

## 3. Results

Parents of 58 patients (116 persons) had been invited to participate in the study. Of these, parents of 11 patients refused to engage in the study; in 4 cases, one parent was assessed and MEFV gene status was not compatible with the patient, and the other parent could not participate in the study. Finally information of 43 patient and their parents was appreciable.

Of the 43 FMF patient, 27 (63%) were male. Mean age at initiation of disease symptoms was 3.4 ± 3.36 years (between 6 months to 20 years old). Eleven patients (26%) had experienced their first complaints near or before their first birthday; 20 (46%) were 1.5 to 4 years old and 7 (16%) at 5 to 7 years old and one patient was at 20 years of age at that time. In 4 cases the parent did not remember the first presentation time.

Nine patients (21%) had homozygote mutation in the MEFV gene and 16 (37%) and 17 (40%) had compound heterozygote and patients with one mutation, respectively. One patient had 3 mutations (complex allele mutations). Frequency of each genotype and mutation in the patients is shown in Table 1, as it was clear that the most homozygote mutation was M694V/M694V and compound heterozygote M694V/R761H, each of them in 4 patients (9%). The most heterozygote mutation in FMF patients was E148Q, in 7 cases (16%).

There was not any significant difference between number of mutation and age of the presentation of the symptoms.

When mutation in MEFV gene was assessed in the parents, most of them were heterozygote, except 2 mothers and 6 fathers, who had compound heterozygote and one father had complex mutations (Tables 2 and 3). Interestingly, the common mutation in nearly all of them was E148Q.  Table 2 also contains mutations of patients according to each allele. It has shown that the most prevalent mutations in both study groups were M694V, E148Q, and M680I.

Fifty-six parents (66%) were of Turk ethnicity origin. The other parents were divided into different races: Turkman, Fars, Kurd, Lor, and Arab. Thirty percent of the parents (parents of 13 patients) were consanguine (second degree relatives). Most of the homozygote patients had consanguine parents (7 of nine patients).

From 43 patients who participated in the study, 41 had mutation(s) similar to their parents. In all of the cases, parents had not any symptoms or complaints regarding the FMF or any other autoinflammatory disease, at the study time or before it, but some of them recalled some nonsignificant vague abdominal pain during their childhood years. In 2 cases, MEFV gene status of patients was different from their parents. The first was a Kurdish boy who had P369S heterozygote mutation, but we could not find any mutation in MEFV gene in both parents, even after rechecking the mutation.

Another patient was a boy, from nonrelative Turk parents. His MEFV gene had compound heterozygote mutation: R761H/M694I. Mutation of father was R761H heterozygote but mother did not have any mutation. The process repeated again, but the same result had been found.

In this study, mutations in MEFV gene were compatible with mutations in fathers in 9 cases (21%), with mothers in 7 (16%), and both of them in 22 (51%). In 2 FMF patients with two mutations, one parent had one of the mutations and the other parent had not been present for assessment. As mentioned before, in two cases there were some discrepancies between the child and her parent (or parents) mutations.

Complex mutations were found if there were more than two mutations in the MEFV gene. We have found a family from north of the country (Turkman ethnicity), in which asymptomatic consanguine parents had more than one mutations, E148Q/P369S and P369S/E148Q/A744S for the mother and father, respectively. The son had complex mutations: E148Q/E148Q/P369S.

Renal amyloidosis complicated a boy with heterozygote mutation of M694V; his healthy mother had the same mutation.

## 4. Discussion

FMF is the most monogenic autoinflammatory disease, inherited autosomal recessively. However, the heterozygote mutation and the patients with only one mutation have usually the FMF disease expressions. [6] In this study, as expected we have found similar mutation in MEFV gene in parents and their offsprings. So, we searched for some exceptions.

In some studies [3, 7], higher frequency of MEFV gene mutation in first-degree relatives of FMF patients had been reported. Lachmann and coworkers [3] reported a group of FMF patients, where 84% of them had at least two mutations and 12% had single mutation. In their study group, 92% of first-degree relatives (parents or siblings) of these patients had mutation in MEFV gene (80% had one mutation, and 12% had two mutations, without any symptoms).

To date, there is not any published article about mutation in both parents of FMF patient specifically; so, against the previous study, the highest percentages (95.3%) of the same MEFV mutation in patients and parents as the first-degree relatives were found.

In exception, the mutations of two FMF patients were not found according to their parents. The patient with single mutation P369S without any mutation in the parents has FMF disease presentation. The P369S is known as polymorphism according to frequency in general population [8], although the role of the allele in inflammatory response to environmental factor as FMF-like disease should not be missed. Also, cooperation between this allele and one or more unidentified modifier alleles could be presented as the typical FMF [9, 10]. So, the polymorphism of P369 and the susceptible races with their related factors such as one or more modifier allele and environmental factors could have this presentation.

The presentation of the disease is correlated with the number of mutation, the patients with more than one mutation express earlier FMF disease presentations [11]; but our study is lacking to show this relationship. This discrepancy may be due to the lower age of the participants in this study.

In this study, the most prevalent mutations in parents and patients are M694V, E148Q and M680I respectively. The result of frequency of FMF mutation in the previous studies of Iran are similar to our study. [12, 13] M694V mutation is the most common mutation in other races such as Turk [14], Jewish [15], and Armenian [16] and less common in Arabs population [17]. In spite of the other populations in the Eastern Mediterranean, E148Q mutations as the second most common mutation with FMF disease presentation have been demonstrated in our study. In Japan [18], where FMF disease is the rare disease, E148Q mutation is the most mutation.

In addition, E148Q/- was found as the most common FMF patients with one mutation. On the other hand, almost all compound heterozygote parents are E148Q/*∗* with any inflammatory presentation. The roles of E148Q mutation in inflammatory presentation are controversy. Although the E148Q variant has the most distribution in the MEFV gene, certain role of this variant remains unclear [19, 20]. In some studies, The E148Q allele with high frequency in both FMF patients and general population has been recognized as polymorphism [19]. In contrast, the hypothesis supports the inflammatory role of E148Q mutation in Turkish and Greek population [21, 22]. So, our study population may have the same specialize environmental or genes.

In almost all of the cases, parents have had mutation(s) similar to their offspring, especially in heterozygote ones, but clinical symptoms are very different, and while a parent with 2 or 3 mutations did not report any symptoms, the child with the same mutation suffered periodic symptoms from early childhood. However, some parents recalled some mild and nonspecific symptoms like abdominal pain during their childhood, just after the result of mutation got clear.

Lachman et al. report higher level of acute phase protein in heterozygous carrier parents in comparison of general population [3].

As mentioned above, the E148Q mutation in almost all healthy compound heterozygous parent are present. Also, the compound heterozygous genotype with E148Q/*∗* allele without any inflammatory symptoms have been reported [3, 23]. On the other hand, when parents have only one mutation E148Q is transmitted to their symptomatic offspring. So, further evaluation about the limiting role of E148Q allele in compound heterozygous genotype in nonsymptomatic population would be involved.

In addition, almost all the offsprings have not been inheriting the E148Q allele from their compound heterozygous parents (Table 3). The similar transmissions of the E148Q allele from compound heterozygous parents to their kids have been reported [7]. In FMF patients, allele may transmit as both autosomal dominant and autosomal recessive pattern. In different population, the autosomal dominant transmission has been reported [24, 25], although the “dose effect” mutation with high penetrance and low penetrance explains the differences mild to more sever presentations [26]. On the other hand, the other factors such as unidentified factor, environmental factors, and epigenetic factors may play role in the pathogenesis of FMF presentations [10]. Furthermore, further expansive evaluation should be considered.

The most of the FMF patients had one or two mutations in MEFV gene, but there are reports of patients with more than 2 mutations in different exons of this gene, so called complex alleles [27, 28]. In this study, such as the others, the combination of two or three allele located on exon 2 (E148Q, L110P) and exon 3 (P639S) can express the FMF phenotype. The cooperation of these two rare exons 2 and 3 may be presented such as exon 10 mutations. In contrast, the exon 2 and exon 3 alleles may only be the polymorphism genotype and the third mutation on exon 10 has function [8, 19]. So, further researches would evaluate this phenomenon.

The frequency of MEFV mutations in the parents of FMF patients was 88%. The exact distributions of MEFV mutation in general population are not available. In a study in Turkish population, the frequency of carrier rate of MEFV gene was 20% [29]. So, we suggest a study for evaluation of the frequency of the MEFV mutation in general population without any symptoms and positive family history of FMF. In addition, evaluation in larger population would be more valuable.

Sometimes periodic fever, aphtous stomatitis, pharyngitis, and cervical adenitis (PFAPA) syndrome mimic FMF symptoms and these patients respond to colchicine. Concurrent and FMF and PFAPA syndrome have been reported frequently [30, 31]. Evidence shows that colchicine is more efficient in PFAPA patients with underlying of MEFV mutation [32–34].

## 5. Conclusion

Most of the patients inherited the mutation in MEFV gen from their parents. The new onset mutation in offspring without mutation in parents is very rare. It is noted that even asymptomatic carriers of mutation in MEFV gene may have some degree of mild inflammation, and close monitoring of parents of FMF patient should be under sought.

## Figures and Tables

**Table 1:** Frequency of different genotype in Familial Mediterranean Fever patients.

	**Genotype**	**Frequency (percent)**
**Heterozygote **17(40)	E148Q	7(16)

	M694V	5(12)

	M680I(G>C)	2(5)

	P369S	1(2)

	V726A	1(2)

	M694I	1(2)

**Compound heterozygote** 16(37)	M694V/R761H	4(9)

	M694I/R761H	2(5)

	M680I/E148Q	2(5)

	M680I/M694V	3(7)

	P369S/E148Q	1(2)

	E148Q/M694I	1(2)

	M694V/V726A	1(2)

	M680I/R761H	1(2)

	V726A/R761H	1(2)

**Homozygote **9(21)	M694V/M694V	4(9)

	M680I/M680I	3(7)

	V726A/V726A	2(5)

**Complex alleles mutations**	P369S/ E148Q/ E148Q	1(2)

**Table 2:** Frequency of mutations in Familial Mediterranean Fever patients and parents.

**Mutation**	**Patients frequency (percent)**	**Parents** **frequency (percent)**
**M694V**	21(30)	19 (25)

**E148Q**	13 (18)	18 (24)

**M680I**	14(20)	13 (17)

**R761H**	8 (11)	8 (11)

**V726A**	7 (10)	7 (9)

**M694I**	4(6)	4 (5)

**P369S**	3(4)	4 (5)

**V744S**		2(3)

**Total number**	70(100)	75(100)

**Table 3:** Genotype of compound heterozygote mutations in the parents.

**Father's genotype**	**ethnicity**	**Mother's genotype**	**Mother's ethnicity**	** Offspring's genotype**
E148Q/M680I	Turk	NA	Fars	M680I

E148Q/M694V	Fars	NA	Fars	M694V

E148Q/P369S	Fars	NA	Fars	E148Q/P369S

E148Q/R761H	Turk	M694V	Turk	M694V/R761H

M694V/A744s	Fars	M694V	Fars	M694V/M694V

E148Q/P369S/A744S	Turkman	E148Q/P369S	Turkman	E148Q/E148Q/P369S

M694V	Turk	E148Q/M694V	Turk	M694V/M694V

NA: not assessed

## Data Availability

The data used to support the findings of this study are available from the corresponding author upon request.
